# Dual-mode solid-state thermal rectification

**DOI:** 10.1038/s41467-020-18212-2

**Published:** 2020-08-28

**Authors:** Ramesh Shrestha, Yuxuan Luan, Xiao Luo, Sunmi Shin, Teng Zhang, Phil Smith, Wei Gong, Michael Bockstaller, Tengfei Luo, Renkun Chen, Kedar Hippalgaonkar, Sheng Shen

**Affiliations:** 1 Department of Mechanical Engineering, Carnegie Mellon University, Pittsburgh, PA 15213 USA; 2 Department of Mechanical and Aerospace Engineering, University of California at San Diego, La Jolla, CA 92093 USA; 3 Department of Aerospace and Mechanical Engineering, University of Notre Dame, Notre Dame, IN 46556 USA; 4 Department of Materials Science and Engineering, Carnegie Mellon University, Pittsburgh, PA 15213 USA; 5 Institute of Materials Research and Engineering, Agency for Science Technology and Research, #08-03, 2 Fusionopolis Way, Innovis, 138634 Singapore

**Keywords:** Devices for energy harvesting, Electrical and electronic engineering, Nanoscale devices

## Abstract

Thermal rectification is an exotic thermal transport phenomenon which allows heat to transfer in one direction but block the other. We demonstrate an unusual dual-mode solid-state thermal rectification effect using a heterogeneous “irradiated-pristine” polyethylene nanofiber junction as a nanoscale thermal diode, in which heat flow can be rectified in both directions by changing the working temperature. For the nanofiber samples measured here, we observe a maximum thermal rectification factor as large as ~50%, which only requires a small temperature bias of <10 K. The tunable nanoscale thermal diodes with large rectification and narrow temperature bias open up new possibilities for developing advanced thermal management, energy conversion and, potentially thermophononic technologies.

## Abstract (web summary)

Here, the authors present a dual-mode solid state thermal rectification effect using a heterogeneous irradiated-pristine polyethylene nanofiber junction as a nanoscale thermal diode, in which heat flow can be rectified in both directions by changing the working temperature.

## Introduction

In an analogy of rectification in electronics, thermal rectification is an important nonlinear and asymmetric thermal transport phenomenon in which heat is regulated to transfer more preferentially in one direction than the other^1–6^. By rectifying heat currents, thermal rectification has substantial implications for thermal transport, for example, by allowing energy systems to dissipate heat to their surroundings but simultaneously protecting them from damage when the surrounding temperature is too high. Although several mechanisms based on thermal expansion^7^, asymmetric nanostructures and mass loading^8–13^, solid–liquid phase change^14,15^, solid-solid phase transition^16–21^ and bi-material interface^22^ were demonstrated for realizing thermal rectification, it has remained a significant challenge to achieve a large and tunable rectification effect, which generally requires either macroscale size or large temperature bias.

Here we demonstrate two types of solid-state nanoscale thermal diodes made from a heterogeneous “irradiated-pristine” polyethylene (PE) nanofiber junction by electron beam (e-beam) irradiation: heavily-irradiated-pristine (HI-P) nanofiber junction and lightly-irradiated-pristine (LI-P) nanofiber junction, which exhibit distinct thermal rectification behaviors. For the HI-P nanofiber junction, the thermal diode can only work around one temperature and rectify heat flow along one direction. In contrast, the LI-P nanofiber junction shows a unique dual-mode thermal rectification effect, which can switch modes to rectify heat flow in both directions by changing the working temperature. For 3 measured HI-P nanofiber samples, we observe an average thermal rectification factor of ~37% and maximum rectification factor of ~50%, which is much higher than the experimental values of nanoscale thermal diodes based on carbon nanotubes (2%)^8^, silicon/polyamide interface (4%)^22^, boron nitride nanotubes (7%)^8^, vanadium dioxide nanobeams (28%)^17^, and graphene (26%)^18^. We also demonstrate a “negative” rectification effect from 3 measured LI-P nanofiber samples, which show an average thermal rectification factor of ~ −12%. The demonstrated thermal rectification can be actively modulated by the environmental temperature and only requires a small temperature bias (<10 K) across the thermal diode.

## Results

### Fabrication of heterogeneous irradiated-pristine nanofiber junctions

**Fig. 1:**
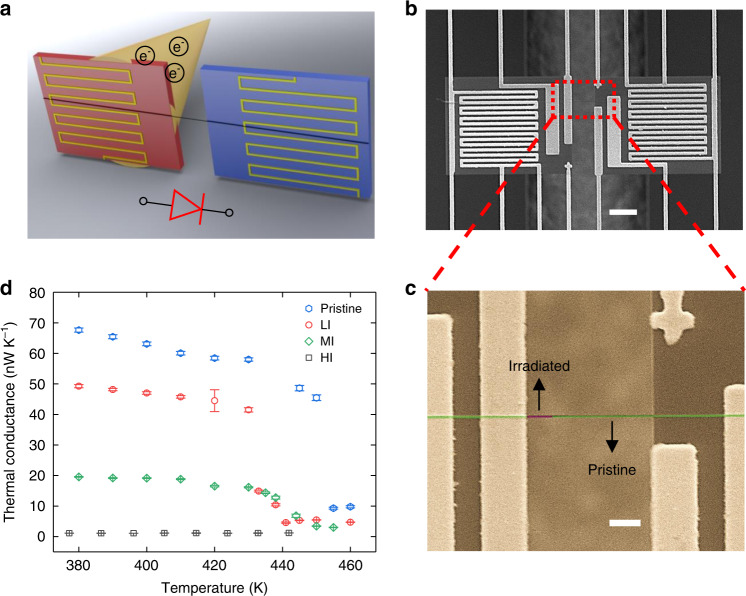
Heterogeneous irradiated-pristine PE nanofiber junction as a nanoscale thermal diode. **a** Schematic and **b** SEM image of the nanofiber junction suspended on the thermometer microdevice consisting of two measurement islands. Scale bar: 10 μm. As illustrated in **a**, one portion of the suspended nanofiber is exposed to e-beam irradiation from the back side of the thermometer microdevice to make the irradiated-pristine nanofiber junction. **c** Zoomed-in SEM image of the nanofiber junction in false colors: purple (irradiated section) and green (pristine crystalline section). Scale bar: 2 μm. **d** Influence of e-beam irradiation on the phase transition behavior of PE nanofibers. The error bar means the standard deviation of thermal conductance data from the Wheatstone measurement.

We fabricate the heterogeneous irradiated-pristine nanofiber junction as a solid-state thermal diode by selectively irradiating one portion of a crystalline PE nanofiber (diameter: 50–200 nm) using an e-beam. As shown by Figs. 1a and b, the PE nanofiber is initially placed on a suspended platinum resistance thermometer microdevice, which consists of a heating and a sensing measurement islands^23,24^. Specifically, by irradiating the nanofiber sample from the back side of the thermometer microdevice via a through-hole in the device (Fig. 1a and Supplementary Fig. 1), the two measurement islands can block the e-beam such that the sections of the nanofiber in contact with the measurement islands remain intact. For the nanofiber junction, 10–20% length of the nanofiber suspended between the two measurement islands is irradiated by the e-beam, as illustrated by the purple region in Fig. 1c, whereas the rest of the fiber is pristine crystalline (green region in Fig. 1c). To measure the thermal rectification effect, the heating island of the thermometer microdevice is heated by a DC current. By applying a small AC current on both the islands to monitor the voltage change, we can measure the temperature difference between the two islands, as well as the heat flow through the nanofiber bridging the islands. In all the measurements, the DC current increases from 0 to 20 µA with a step of 0.2–0.4 µA. At each step, we collect the experimental data after the whole device reaches thermal equilibrium.

The observed thermal rectification effect arises from the unique thermal switching behavior in pristine crystalline PE nanofibers occurring around the phase transition temperature (see Supplementary Note 2). Crystalline PE nanofibers have an anisotropic molecular structure with very strong carbon-carbon covalent bonds along the polymer chain but weak van der Waals bonds between the chains. At low temperatures, the nanofibers have a high thermal conductivity along the fiber direction due to the highly ordered and aligned chains^25,26^. However, beyond a threshold temperature for overcoming the dihedral angle energy barrier, random segmental rotations occur along the polymer chains, leading to a much lower thermal conductivity. Such a dramatic change in morphology (Supplementary Fig. 2) also indicates a structural phase transition from a highly ordered conformation (i.e., high thermal conductivity phase) to a rotationally disordered one (i.e., low thermal conductivity phase)^27–29^. As shown by the blue circles in Fig. 1d, the measured thermal conductance of a pristine crystalline PE nanofiber first gradually decreases with temperature as explained by the well-known Umklapp scattering of phonons in crystals and then abruptly drops from ~45 nW K^−1^ to ~9 nW K^−1^ around 450 K due to the structural phase transition^27^. This sharp change of thermal conductance corresponds to a thermal switching ratio *f* ~ 5, where *f* = *G*_on_/*G*_off_ is defined as the ratio of the on-state high thermal conductance *G*_on_ to the off-state low thermal conductance *G*_off_.

We further tune the phase transition temperature and the thermal switching ratio of the nanofibers by e-beam irradiation, which reduces both their molecular orientation and crystalline domain^30,31^, as confirmed by the micro-Raman measurements in Supplementary Fig. 3 and Table 1. With a low accelerating e-beam voltage in a scanning electron microscope (SEM), we image and irradiate the entire previously measured pristine nanofiber for ~2 s. Compared to the pristine nanofiber, the measured thermal conductance of the lightly irradiated (LI) nanofiber decreases in the whole temperature range considered (380–460 K) due to the reduced molecular orientation and crystallinity, and the phase transition temperature is shifted from ~450 K to ~430 K, as illustrated by the red circles in Fig. 1d. The thermal switching ratio of the LI nanofiber increases to *f* ~9, higher than that of the pristine nanofiber (*f* ~ 5), due to the lower *G*_off_ in the LI nanofiber. When irradiating the nanofiber sample for an additional ~2 s (i.e., ~4 s in total), the moderately irradiated (MI) nanofiber displays a smoother thermal switching behavior than those of the LI and the pristine ones, and its switching ratio is reduced to *f* *~* 3.7, as shown by the green diamonds in Fig. 1d. Yet the phase transition temperature of the MI nanofiber remains almost the same as that of the LI one. With more intense e-beam irradiation (e.g., >6 s in total), the heavily irradiated (HI) nanofiber has no apparent phase transition and exhibits a slightly increasing thermal conductance trend like that of an amorphous material (black squares in Fig. 1d and Supplementary Fig. 10).

### Thermal rectification mechanisms of HI-P and LI-P nanofiber junctions

**Fig. 2:**
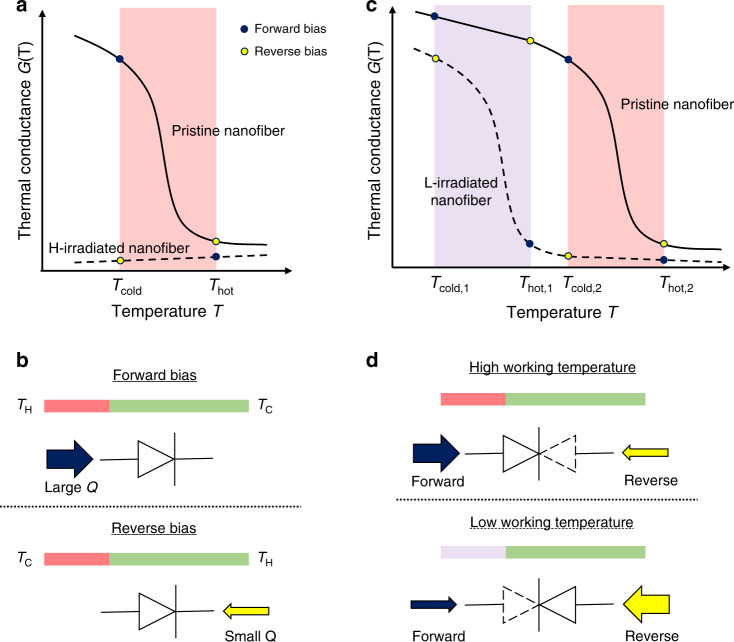
Schematics of thermal rectification behaviors of HI-P and LI-P nanofiber junctions. **a** and **b** HI-P nanofiber junction showing single-direction rectification due to the phase transition of the pristine segment. **c** and **d** LI-P nanofiber junction showing dual-mode thermal rectification at different working temperatures. At the high working temperature, the phase transition of the pristine segment induces a rectification behavior like that of the HI-P junction. At the low working temperature, the phase transition of the LI segment gives rise to the rectification in the opposite direction. The pink region in **a** and **c** represents the scenario that heat flow in the reverse bias is suppressed and the junction shows positive rectification. The purple region in **c** represents the scenario of the LI-P nanofiber junction that heat flow in the forward bias is suppressed and the junction shows negative rectification. The solid and open circles in **a** and **c** mark different working states under forward or reverse bias.

In Figs. 2a and c, based on the controlled phase transition and thermal switching (Fig. 1d) by e-beam irradiation, we design two types of solid-state nanoscale thermal diodes with distinct thermal rectification behaviors: heavily-irradiated-pristine (HI-P) nanofiber junction and lightly-irradiated-pristine (LI-P) nanofiber junction. For the HI-P nanofiber junction, the resulting thermal diode can only work around one temperature and rectify heat flow along one direction. In the forward temperature bias (Fig. 2b), where the pristine segment is on the cold side (*T*_cold_ in Fig. 2a), the pristine segment does not undergo phase transition and maintains a high thermal conductance. Therefore, the overall thermal conductance of the HI-P nanofiber junction is higher, which renders larger heat transfer from left to right (Fig. 2b). When the temperature bias is reversed, the higher temperature (*T*_hot_ in Fig. 2a) in the pristine segment induces the phase transition resulting in a smaller heat flow from right to left (Fig. 2b). In both the biasing conditions, the thermal conductance of the HI segment has a minimal change (black squares in Fig. 1d and dashed line in Fig. 2a). The HI-P nanofiber junction thus performs as a regular thermal diode with a larger heat flow in the forward bias than that in the reverse bias.

In contrast, the LI-P nanofiber junction shows a special dual-mode thermal rectification effect, in which heat flow can be rectified in both directions depending on the working temperature. As shown by the purple and the pink regions in Fig. 2c, the LI and the pristine segments in the LI-P nanofiber junction, respectively, undergo the phase transition at different temperatures. For the LI-P nanofiber junction, we define the forward bias from the LI segment to the pristine segment, which is consistent with the one for the HI-P nanofiber junction. In Fig. 2d, at a high working temperature around the phase transition of the pristine segment (pink region in Fig. 2c), the heat flow across the LI-P nanofiber junction in the forward bias is larger than that in the reverse bias, similar to the case in the HI-P nanofiber junction. However, at a low working temperature around the phase transition of the LI segment (purple region in Fig. 2c), the thermal transport in the reverse bias surpasses the one in the forward bias, rectifying heat flow in the opposite direction. This is because in the forward bias, *T*_hot,1_ induces the phase transition in the LI segment and therefore leads to a low thermal conductance.

### Demonstration of HI-P nanofiber junctions as single-mode nanoscale thermal diodes

**Fig. 3:**
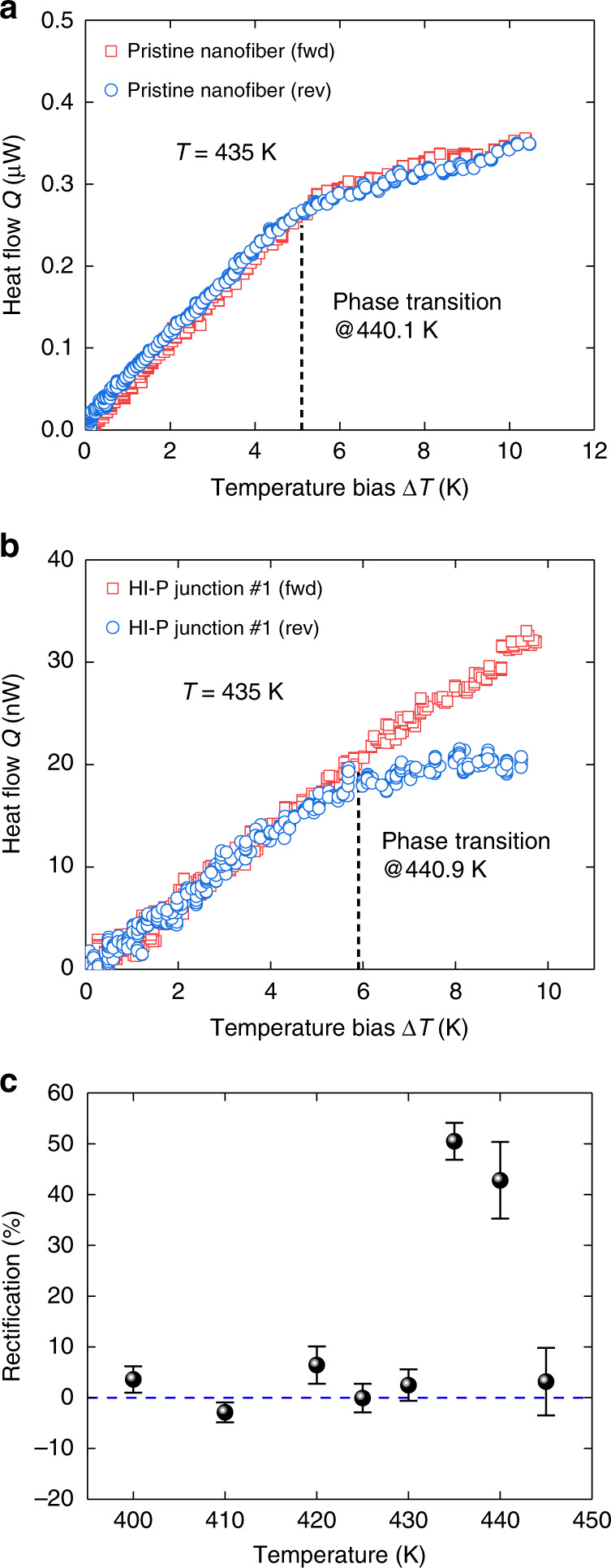
Measured thermal rectification of HI-P nanofiber junction #1. **a** Heat flow of a pristine crystalline PE nanofiber as a function of temperature bias Δ*T* under the forward and reverse biases at environmental temperature *T* = 435 K. **b** Heat flow of HI-P junction #1 under the forward and reverse biases at environmental temperature *T* = 435 K. The black dashed lines mark the phase transition temperatures. **c** Thermal rectification factor *R* as a function of the environmental temperature with the fixed temperature bias Δ*T* = 9 K. The blue dashed line is for eye guidance of zero rectification. The error bar of rectification is calculated from least-square fitting of the heat flow curve, which is elaborated in Supplementary Note 8.

We selectively irradiate 10–20% length of the nanofibers to fabricate the HI-P and the LI-P nanofiber junctions in order to maximize their thermal rectification performance (see Supplementary Figs. 4 and 5 for theoretical optimization). As a reference experiment, we first measure the heat flow *Q* of a pristine crystalline PE nanofiber as a function of temperature bias Δ*T* in the forward (*Q*_fwd_) and reverse (*Q*_rev_) directions at an environmental temperature of 435 K. In Fig. 3a, the heat flows in the two directions essentially overlap at the same temperature, indicating no rectification effect. The apparent non-linearity in the *Q* vs. Δ*T* curves in Fig. 3a is due to the thermal conductance change as the phase transition occurs. For HI-P nanofiber junction #1 (Fig. 3b), however, *Q*_fwd_ is clearly higher than *Q*_rev_ when Δ*T* > 5 *K*, which corresponds to the onset of phase transition in the pristine segment. Fig. 3c shows the thermal rectification factor *R* as a function of the environmental temperature for the temperature bias Δ*T* = 9 K, where *R* is defined as *R* = (*Q*_fwd _ – *Q*_rev_)/*Q*_rev_. Even with such a small temperature bias (Δ*T* = 9 K), a peak thermal rectification factor *R* = 50.5 ± 3.6% is achieved around 435 K. For HI-P nanofiber junction #2, we observe the thermal rectification factor to be 48.2 ± 0.3% at Δ*T* = 10K, *T* = 435 K (Supplementary Fig. 6a). For HI-P nanofiber junction #3, its thermal rectification factor is 13.8 ± 0.6% at Δ*T* = 7K, *T* = 448 K (Supplementary Fig. 6b). The variation of rectification factors is mainly attributed to the thermal switching ratio of pristine crystalline nanofibers, which usually ranges from 5 to 10.^27^

### Dynamic responses of HI-P nanofiber junctions upon multiple cycles

**Fig. 4:**
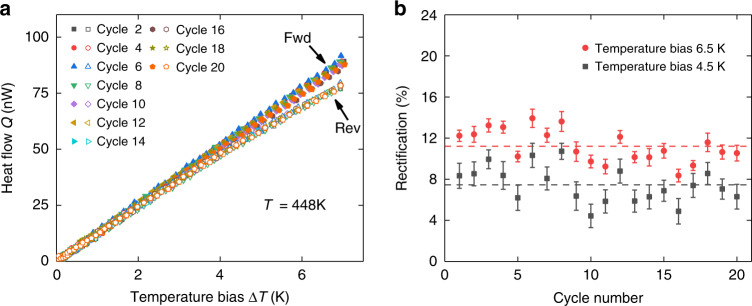
Dynamic response of HI-P nanofiber junction #3 at *T* = 448 K. **a** Heat flow vs. temperature bias for even-number cycles. Solid and hollow signs indicate the forward and reverse directions, respectively. **b** Rectification factor vs. cycle number at 4.5 K and 6.5 K temperature biases. Dashed lines show the average rectification values, which are 7.5% and 11.2% at 4.5 K and 6.5 K temperature biases, respectively. The error bar of rectification is calculated from least-square fitting of the heat flow curve, which is elaborated in Supplementary Note 8.

To evaluate the dynamic response of the nanoscale thermal diodes, we first measure the temperature-dependent thermal rectification factors of HI-P nanofiber junction #3 by sweeping the environmental temperature until a pronounced thermal rectification effect (rectification factor >10%) is observed at the temperature of 448 K. Then, we anneal and stabilize HI-P nanofiber junction #3 by holding its environmental temperature at 448 K for 6.5 h. After that, we thermally cycle the sample for 20 times by alternately switching its temperature bias from the forward direction to the reverse direction. In Fig. 4a, we plot the heat flows as a function of the temperature bias for both the forward and reverse directions. In the forward bias, the pristine segment of HI-P nanofiber junction #3 is on the cold side and thus phase transition does not occur. The heat flow vs. temperature bias curves are almost linear. However, for a reversed bias, the pristine portion is on the hot side and the resulting phase transition causes the heat flow vs. temperature bias curves to bend downward, which corresponds to a continuous decrease of thermal conductance. In Fig. 4b, the measured thermal rectification factors are plotted as a function of cycle numbers at temperature biases of 4.5 K and 6.5 K, respectively. Within 20 cycles, the rectification values in average are 7.5% with 1.7% standard deviation at 4.5 K temperature bias, and 11.2% with 1.6% standard deviation at 6.5 K temperature bias, respectively.

### Demonstration of LI-P nanofiber junctions as dual-mode nanoscale thermal diodes

**Fig. 5:**
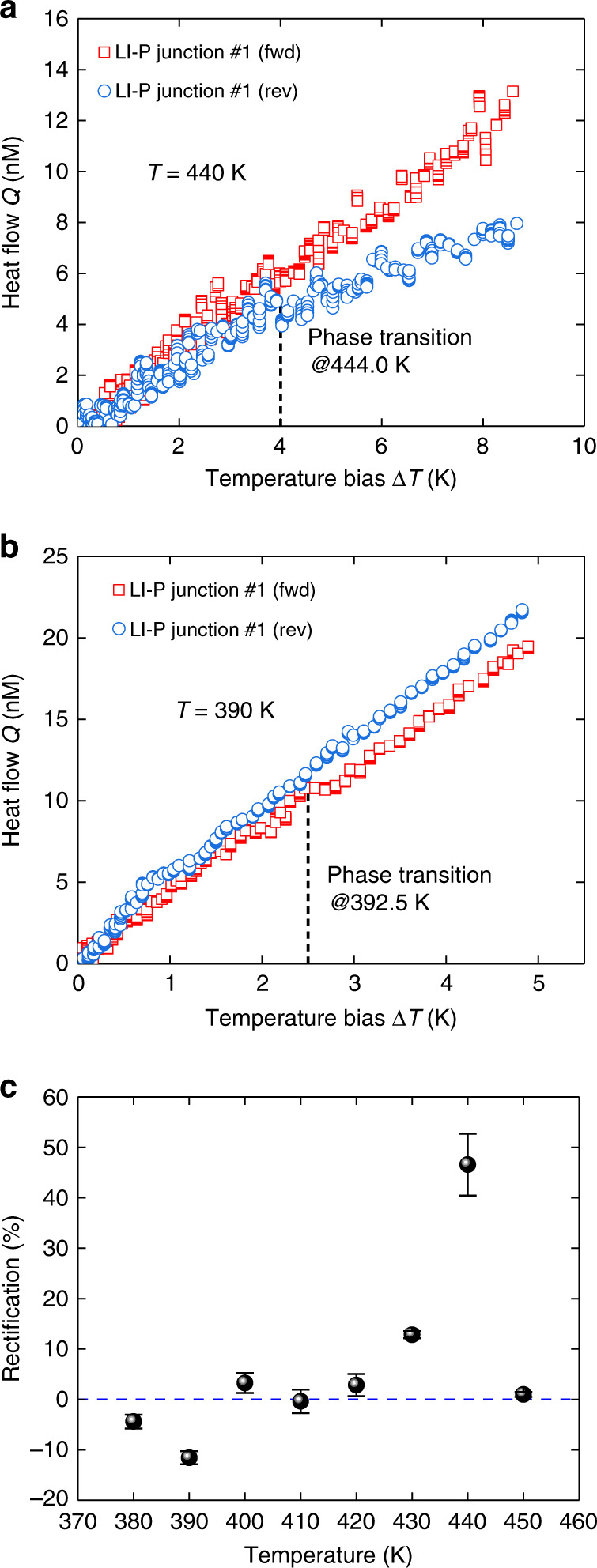
Measured dual-mode rectification of LI-P nanofiber junction #1 at different working temperatures. **a** and **b** Heat flow of LI-P nanofiber junction #1 under the forward and reverse biases at environmental temperatures *T* = 440 K and *T* = 390 K, respectively. At a higher working temperature in **a**, LI-P nanofiber junction #1 shows positive rectification. At a lower working temperature in **b**, LI-P nanofiber junction #1 shows negative rectification, i.e., heat flow in the forward direction is lower than that in the reverse direction. **c** Dual-mode rectification as a function of environmental temperature. The thermal rectification factor *R* is calculated with temperature biases Δ*T* that are 3 K and 2 K over the phase transition temperatures in **a** and **b**, respectively. The error bar of rectification is calculated from least-square fitting of the heat flow curve, which is elaborated in Supplementary Note 8.

For LI-P nanofiber junction #1, *Q*_fwd_ is higher than *Q*_rev_ at a relatively high working temperature (e.g., *T* = 440 K), which is attributed to the higher phase transition temperature of the pristine segment (Fig. 5a). Yet, at a relatively low temperature (e.g., *T* = 390 K) where the LI segment undergoes the phase transition, *Q*_rev_ exceeds *Q*_fwd_ such that the calculated *R* becomes “negative” (Fig. 5b). In Fig. 5c, compared with HI-P nanofiber junction #1, LI-P nanofiber junction #1 shows one rectification peak and one rectification valley around 440 K and 390 K, respectively, where the peak *R* = 46.6 ± 6.1% is positive but the valley *R* = −11.6 ± 1.3% is negative. As the repeated samples, we observe *R* = −11.5 ± 2.9% and *R* *=* −13.7 ± 3.5% for LI-P nanofiber junctions #2 and #3, respectively (see Supplementary Fig. 7). Hence, with LI-P nanofiber junctions, we achieve a tunable dual-mode thermal rectification effect that can selectively block heat flow in a certain direction and work at different temperature ranges.

## Discussion

The rectification performance of the thermal diodes directly relies on the initial properties of pristine PE nanofibers, particularly, the thermal switching ratio. As suggested by molecular dynamics study^28^, strain can help restore the highly ordered phase at low temperatures, and thus the thermal switching ratio varies with the strain of the PE nanofiber. Due to the limitation of our current fabrication, the thermal switching ratio of pristine PE nanofibers and further the rectification ratio of the thermal diodes are sample dependent. We believe that the consistency of the current experiment can be improved if scalable fabrication is developed with accurate control of fiber diameter and stress.

In summary, by fabricating the irradiated-pristine junction as a nanoscale thermal diode, we demonstrate dual-mode solid-state thermal rectification in which heat flow is rectified from either the forward or the reverse direction depending on the working temperature. This unusual thermal transport phenomenon roots from the controlled phase transition and thermal switching of crystalline PE nanofibers enabled by e-beam irradiation. For 6 nanofiber samples, the maximum rectification factor *R* ~ 50% is achieved for the nanoscale thermal diodes developed in this work, exceeding the experimentally reported values of existing solid-state nanoscale thermal diodes based on carbon nanotubes, silicon/polyamide interface, boron nitride nanotubes, graphene and vanadium dioxide nanobeams. The dual-mode thermal rectification demonstrated in this work can potentially be utilized to actively regulate heat flow for advanced thermal management and energy conversion. Currently, the thermal diodes only work around the phase transition temperature of PE nanofibers. Phase transition temperature decreases with weaker dihedral angle strength and interchain interaction^32^. The dual-mode thermal rectification demonstrated in this work can be extended to a wider temperature range if the phase transition temperature is chemically tuned.

## Supplementary information

Supplementary Information

## Data Availability

The data that support the findings of this study are available in Supplementary Information and from the corresponding authors upon request.

## Code availability

The MATLAB codes that support the findings of this study are available from the corresponding authors upon request.
