# Supplementary material for: Dual-mode solid-state thermal rectification
Source: Nat Commun. 2020 Aug 28;11:4346. doi: 10.1038/s41467-020-18212-2 (PMC7455708; doi:10.1038/s41467-020-18212-2)
Supplement: Supplementary file 1 — Supplementary Information [file 41467_2020_18212_MOESM1_ESM.pdf]

Supplementary Information for

Dual-Mode Solid-State Thermal Rectification

Ramesh Shrestha, Yuxuan Luan, Xiao Luo, Sunmi Shin, Teng Zhang, Phil Smith, Wei Gong, Michael Bockstaller, Tengfei Luo, Renkun Chen, Kedar Hippalgaonkar, Sheng Shen†

†Corresponding author. Email: sshen1@cmu.edu.

Supplementary Note 1: Fabrication of the thermal diodes based on PE nanofibers.

To prepare a PE gel for processing nanofibers, PE powder (average molecular weight: $3-6\ \times 10^6$ g mol^{-1}, Sigma Aldrich) is mixed with decalin (Sigma Aldrich) and gets heated up to 145 ℃ to form a transparent and viscous solution with 0.8 wt % PE. The heating process is implemented in an argon filled glove box to avoid oxidation and degradation. Then PE gel is formed by quenching the solution to the room temperature in a water bath. The PE nanofibers are processed through a two-step process. First, a ~ 1cm long PE microfiber is drawn from the PE gel by a sharp glass tip (10 µm in diameter) at the ambient temperature of around 90 ℃. Next, this PE microfiber is collected and stored under tensile stress by a sample collector – a bulk micro-machine silicon frame with a square hole. The pre-stressed PE microfiber is locally heated near the melting point by a tungsten micro heater and gets scaled down to a nanofiber with a diameter in the range of 50 nm - 200 nm. Finally, the PE nanofiber is tightly fixed on the suspended thermometer device by capillary-assisted adhesion, where an isopropanol droplet is applied onto the device before placing

the nanofiber.

To fabricate a thermal diode, we use e-beam to irradiate the suspended portion of a pristine crystalline PE nanofiber across two measurement islands. The suspended length is imaged and irradiated through FEI Quanta 600 FEG Scanning Electron Microscope under an accelerating voltage of 5 kV. For LI-P nanofiber junction it is irradiated for ~2 s and for HI-P nanofiber junction it is irradiated for >6 s. Specifically, we irradiate the nanofiber from the back side of the thermal device (Supplementary Fig. 1) in order to protect the sections of the nanofiber on the measurement islands from the e-beam. By irradiating the nanofiber from the backside, the influence of contact resistance on the performance of the thermal diode can be minimized so that the thermal contact resistance maintains the same in both the forward and reverse thermal biases. This ensures that the observed rectification is mainly caused by the heterogeneous irradiated-pristine junction where the two materials in the junction have different temperature-dependent thermal conductance.

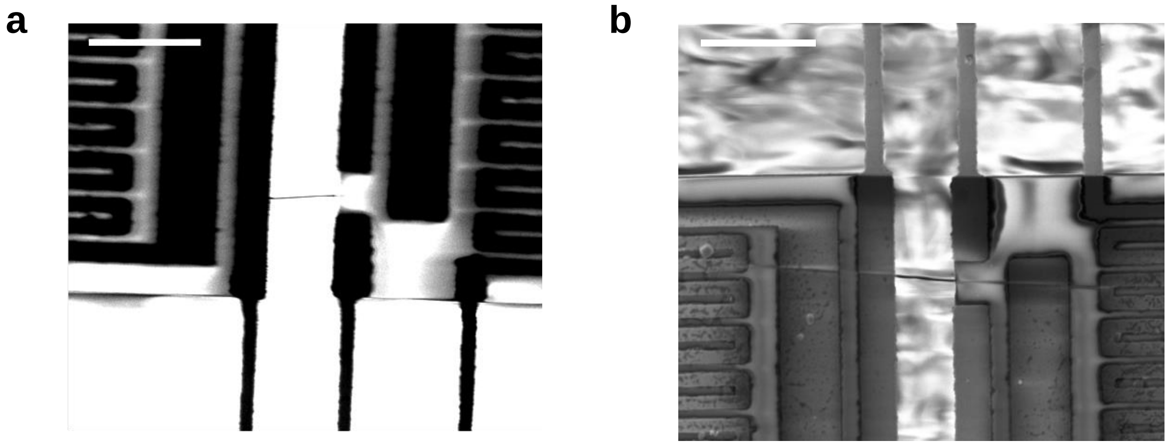

Supplementary Figure 1: Comparison of the SEM images of a suspended PE nanofiber from (**a**) the back-side view and (**b**) the front-side view. When irradiating the nanofiber from the backside of the thermal device, the

measurement islands can block the e-beam and thus protect the sections of the nanofiber on the measurement islands. The scale bar is 10 μm.

Supplementary Note 2: Phase transition of PE nanofibers

As seen in Supplementary Fig. 2a, the PE nanofibers have an orthorhombic crystal structure before the phase transition. Due to its anisotropic molecular structure where there are strong carbon-carbon bonds along the chain but relatively weak dihedral angle energy and inter-chain van der Waals interaction, these PE chains are much stiffer in terms of the bond stretching than the bond rotation. As a result, the PE nanofibers have an intrinsic high thermal conductivity along the chain direction due to the aligned and highly ordered carbon segmental arrangement [1,2].

However, when temperature increases to the phase transition temperature, allowing the atomic kinetic energy to overcome the weak dihedral energy barrier, segmental rotations of PE chains occur, which introduces a structural phase transition from a highly ordered *all-trans* conformation to a combined *trans* and *gauche* conformation with rotational disorder (Supplementary Fig. 2b) [3-6]. Such a dramatic change in morphology can drastically induce phonon scattering along the chains, resulting in a low thermal conductivity after the phase transition.

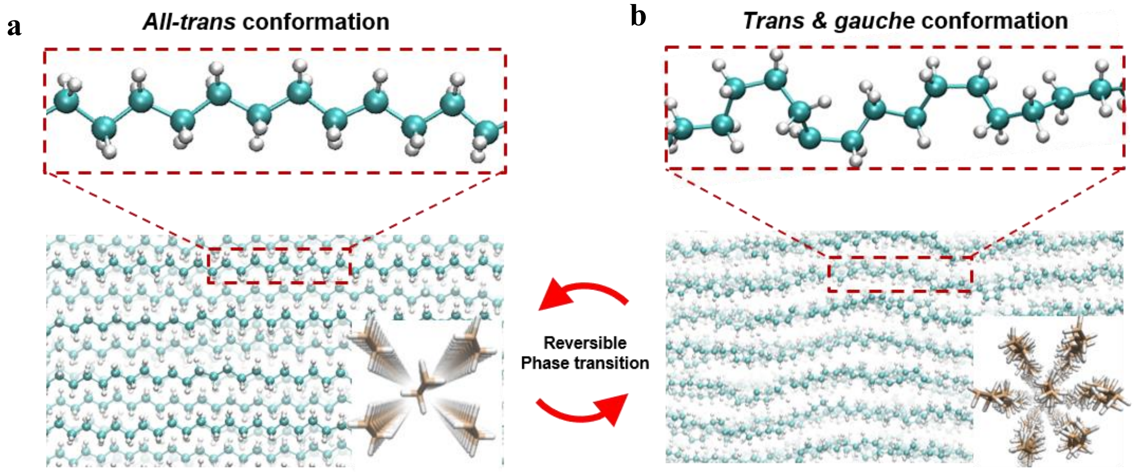

Supplementary Figure 2: Schematics of the molecular structure of the PE nanofibers (**a**) before the phase transition and (**b**) after the phase transition. Insets: molecular structures in the cross section of the nanofibers.

Supplementary Note 3: Raman micro spectroscopy of PE powder, irradiated PE nanofiber and pristine PE nanofiber

To investigate the influence of e-beam irradiation on PE nanofibers, we performed Raman measurements on PE powder, irradiated PE nanofiber and pristine PE nanofiber. As seen in Supplementary Fig. 3, based on the Ref. [7], there is a broad peak at 1080 cm^{-1} which represents the gauche conformation in the amorphous phase and a peak at 1170 cm^{-1} which represents the methylene rocking modes of the crystalline and amorphous phases in the PE powder Raman micro spectroscopy. In comparison, the Raman micro spectroscopy of the PE nanofiber shows the absence of these peaks. Moreover, several peaks at 1430-1460 cm^{-1} which represent methylene deformation modes disappear in the pristine PE nanofiber Raman spectra. In addition to the absence of peaks, the intensities of peaks are different in the Raman measurements of the PE

powder and the pristine PE nanofiber. In the pristine PE nanofiber Raman spectra, the peak intensity at 1296 cm^{-1} representing the methylene twisting modes of the crystalline phase is smaller, and the peak intensity at 1418 cm^{-1} which is the characteristic of the orthorhombic crystalline phase is larger. All these differences demonstrate that the pristine PE nanofiber has a much higher degree of crystallinity and more aligned molecular orientation than PE powder. After the e-beam irradiation, the peak intensities at 1060 cm^{-1} (C-C antisymmetric stretching modes), 1170 cm^{-1} and 1430-1460 cm^{-1} are higher than those of the pristine PE nanofiber. As shown in Supplementary Table 1, the decrease of the peak intensity ratio I_{1128}/I_{1060} and the integral area $A_{1414}/A_{1293+1305}$ indicates that molecular chains are less oriented and that the crystallinity is lower than before, respectively. This is the reason why the irradiated PE nanofiber has a different temperature-dependent thermal conductance compared to the pristine PE nanofiber.

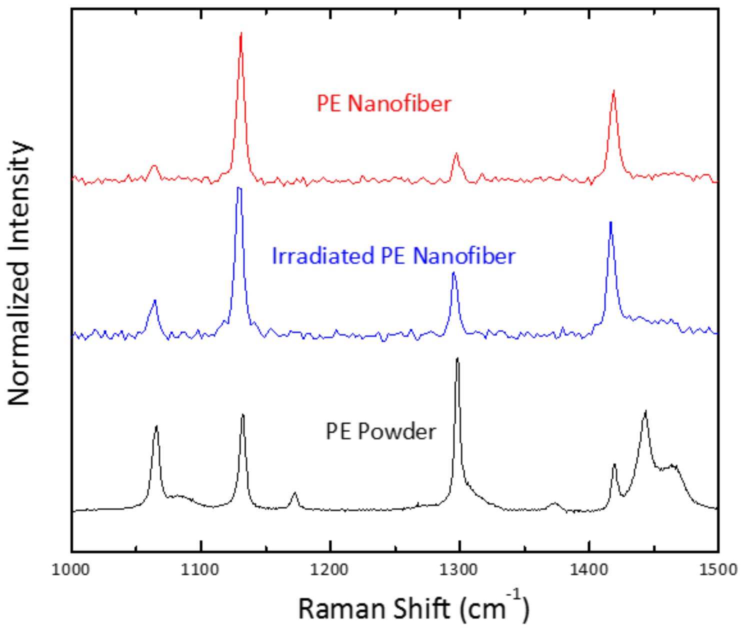

Supplementary Figure 3: Comparison of the Raman micro-spectroscopy of PE Nanofiber, irradiated PE nanofiber and pristine PE nanofiber.

Test sample	PE powder	Irradiated PE nanofiber	Pristine PE nanofiber
I_{1128}/I_{1060}	1.2	3.6	7.8
$A_{1414}/A_{1293+1305}$	0.2	2.5	4.4

Supplementary Table 1: Ratio of peak intensity and integrated area of typical peaks. The first row shows different kinds of the test samples – PE powder, irradiated PE nanofiber and pristine PE nanofiber. The second and the third rows show the peak ratio of I_{1128} and I_{1060} and the integrated area of A_{1414} and $A_{1293+1305}$ in different samples, respectively.

Supplementary Note 4: Theoretical Analysis of the performance of the nanoscale thermal diodes

Here we adopted the 1D thermal diode model developed by Ref. [8] to investigate the parametric influence on the rectification performance. As discussed in Section 7, the thermal contact resistance between the nanofiber and the islands is much smaller than the thermal resistance of the suspended nanofiber. Therefore, the thermal contact resistance is assumed to be negligible in this theoretical model. The thermal conductance of both irradiated and pristine materials in unit length is defined as a piecewise function consisting of three power functions with different power values:

$$G(T) = \begin{cases} G_{on}\left(\frac{T}{T_1^*}\right)^{n_1} & T < T_1^* \\ G_{on}\left(\frac{T}{T_1^*}\right)^{n_t} & T_1^* < T < T_2^* \\ G_{off}\left(\frac{T}{T_1^*}\right)^{n_2} & T > T_2^* \end{cases} \qquad (S1)$$

where phase transition begins at T_1* and ends at T_2*, and G_{on} and G_{off} are the thermal conductance right before and after phase transition, respectively. Temperature values T_1* and T_2*, thermal conductances G_{on} and G_{off} and power *n* are given based on the experimental data of our measurement in Ref. [9] and Fig. 1d. As a simplification, the thermal conductance of the HI nanofiber is assumed to be a temperature-independent constant, which is 1 nW K^{-1}. As shown in Fig. 4, rectification value is affected by the length fraction of the irradiated segment, and switch factors of the pristine PE nanofiber for the HI-P nanofiber junction and the LI nanofiber for the LI-P nanofiber junction, respectively. Here, our results show that the optimal irradiated length fractions for the positive rectification in HI-P nanofiber junction and the negative rectification in LI-P nanofiber junction should be in the range of 5 ~ 20 %. For the HI-P nanofiber Junction, the heat flow as a function of temperature bias is shown in Fig. 5a for the forward (solid lines) and reverse (dashed lines) biases. Figures 5b and 5c show the heat flow vs. temperature bias curves for the LI-P nanofiber Junction at high and low environmental temperatures, respectively. A positive rectification effect occurs at a high environmental temperature, whereas a negative rectification is observed at a low environmental temperature. The environmental temperature also influences the rectification value of the thermal diodes.

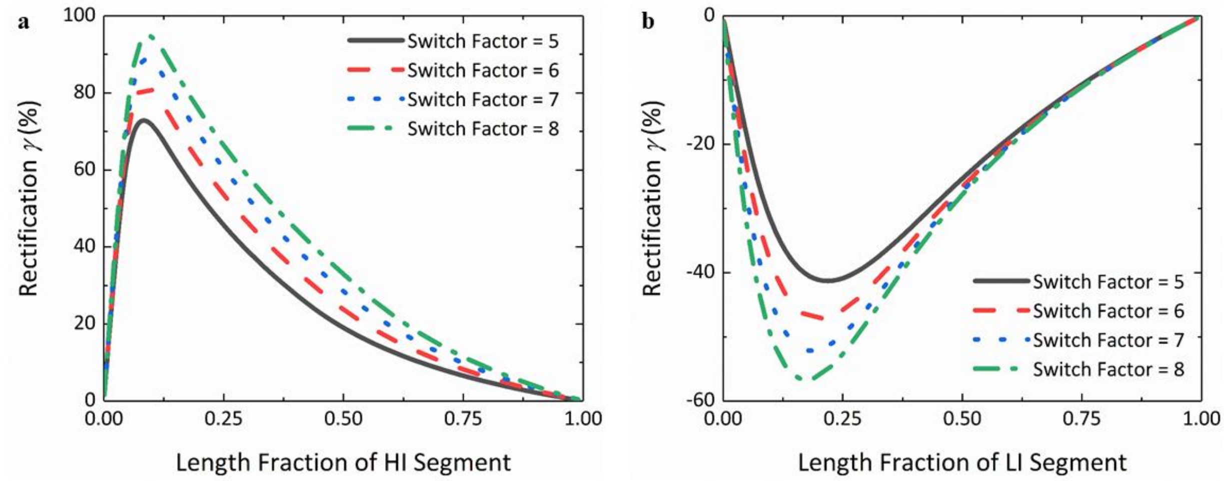

Supplementary Figure 4: Influence of the length fraction of irradiated segment on the rectification value in (**a**) the HI-P nanofiber junction with different switch factors of the pristine segment and (**b**) the LI-P nanofiber junction with different switch factors of the LI segment.

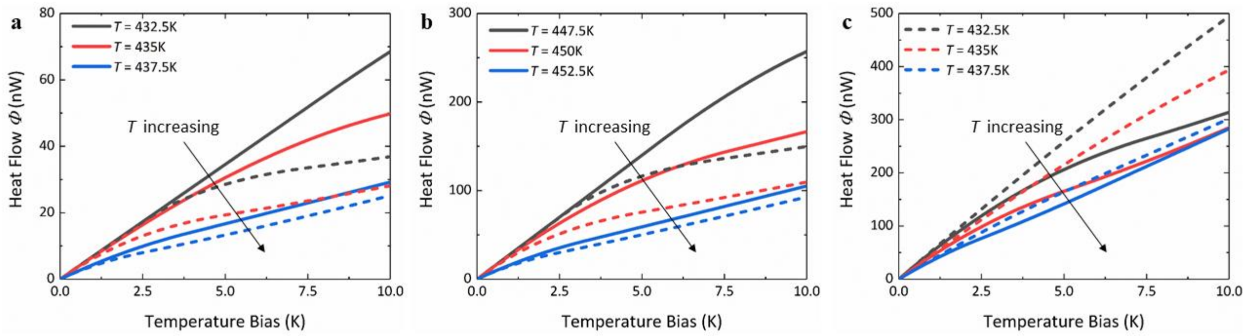

Supplementary Figure 5: Heat flow versus temperature bias at different working temperatures: (**a**) HI-P nanofiber junction, (**b**) LI-P nanofiber junction at a high working temperature, and (**c**) LI-P nanofiber junction at a low working temperature. Solid and dash lines represent heat flows in the forward bias and the reverse bias cases, respectively.

Supplementary Note 5: Rectification of HI-P nanofiber junctions #2 and #3

For HI-P nanofiber junction #2, a strong thermal rectification effect is observed at the environmental temperature of 435 K. As shown in Supplementary Fig. 6, phase transition of the pristine portion occurs at 437 K and consequently the heat flow curve in the reversed bias bends down as the temperature bias is greater than 2 K while the heat flow curve in the forward bias maintains linear, indicating an almost constant thermal conductance. As a result, HI-P nanofiber junction #2 shows 48.2 ± 0.3% rectification at 10 K temperature bias. In Supplementary Fig. 6b, HI-P nanofiber junction #3 shows 13.8 ± 0.6% rectification at 7 K temperature bias and the environmental temperature of 448 K. We also use HI-P nanofiber junction #3 for cycling tests (Fig. 4). Here, Supplementary Fig. 6b shows the results of the first cycle.

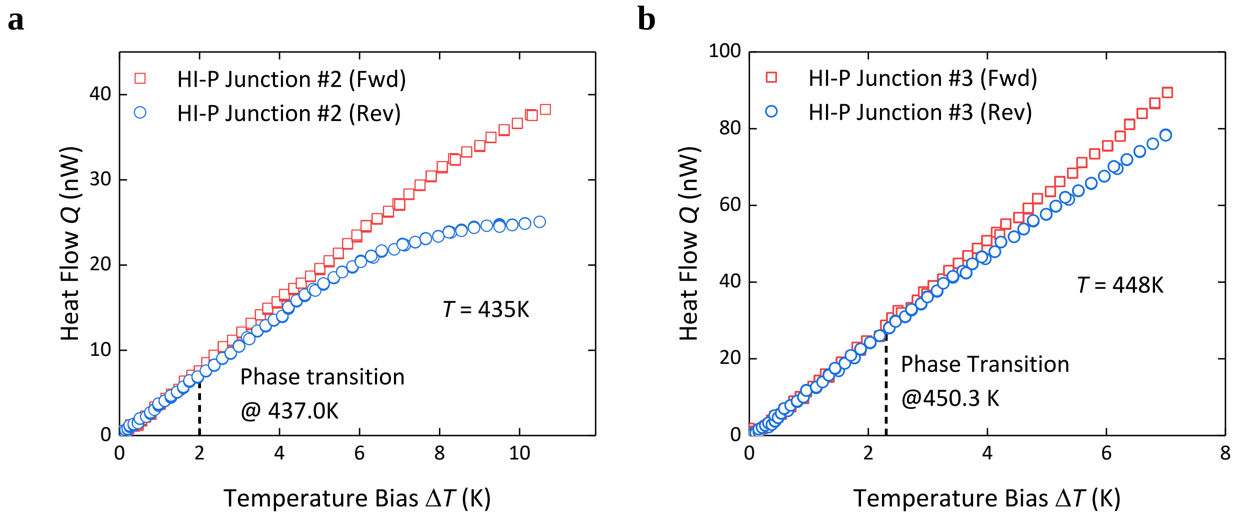

Supplementary Figure 6: Measured rectification of HI-P nanofiber junctions #2 and #3. (**a**) Heat flow vs. temperature bias curves of the HI-P nanofiber junction #2 under the forward and reverse biases at temperature T = 435 K. (**b**) Heat flow vs. temperature bias curves of HI-P nanofiber junction #3 under the forward and reverse biases at temperature T = 448 K.

Supplementary Note 6: Rectification of LI-P nanofiber junctions #2 and #3

For LI-P nanofiber junction #2 (Supplementary Fig. 7a), phase transition of the LI portion occurs at 396.3K and rectification factor is −11.5 ± 2.9% at 4 K temperature bias. For LI-P nanofiber junction #3 (Supplementary Fig. 7b), phase transition of the LI portion occurs at 410.5 K and rectification factor is −13.7 ± 3.5% at 8.5 K temperature bias.

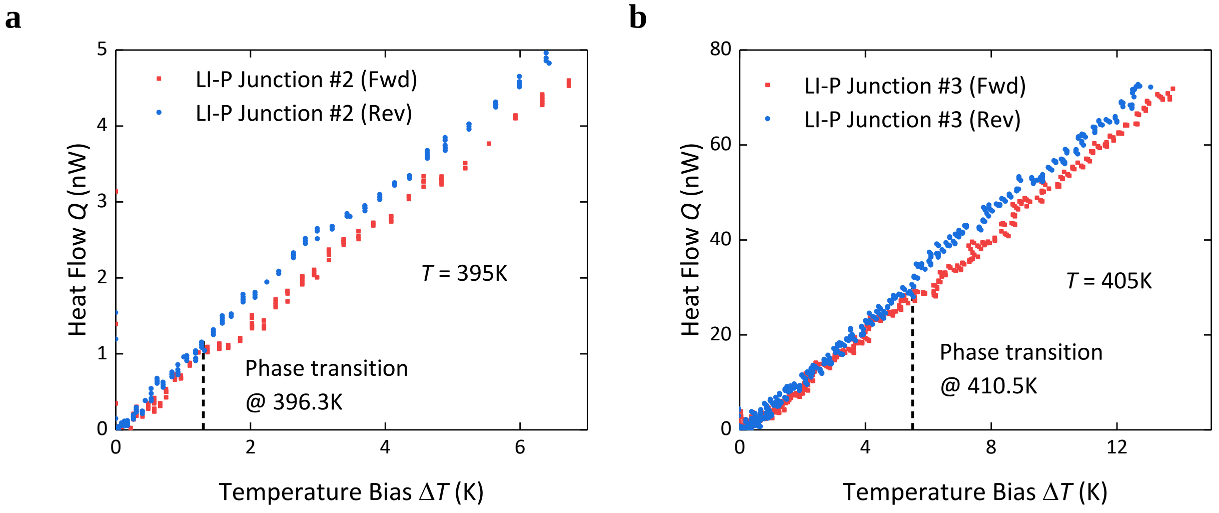

Supplementary Figure 7: Measured negative rectification of LI-P nanofiber junctions #2 and #3. (**a**) Heat flow vs. temperature bias curves of the LI-P nanofiber junction #2 under the forward and reverse biases at temperature $T = 395$ K. (**b**) Heat flow vs. temperature bias curves of LI-P nanofiber junction #3 under the forward and reverse biases at temperature $T = 405$ K.

Supplementary Note 7: Thermal contact resistance between the measurement islands and the nanofiber

To reduce the thermal contact resistance between the measurement islands and the nanofiber, we

employ the capillary-assisted adhesion by placing an isopropanol droplet on top of the microthermal device when mounting the nanofiber. The approximation of the thermal contact resistance is calculated based on the analysis in Ref. [10] and the line contact model developed in Ref. [11]. In Supplementary Fig. 8, the thermal contact resistance R_c between a ~ 100 nm thick PE nanofiber and suspended islands is around 5.5×10^6 KW^{-1} to 6.2×10^6 KW^{-1} before the phase transition. This value is consistent with the measured value in Ref. [2]. After the phase transition, the thermal contact resistance increases up to around 11.1×10^6 KW^{-1} to 19.2×10^6 KW^{-1} due to the drop in the thermal conductance of the PE nanofiber. According to our thermal measurements, the thermal conductance of a ~ 100 nm thick PE nanofiber is on the order of 10 nW/K and 1 nW/K before and after the phase transition, respectively, corresponding to the thermal resistances of 10^8 KW^{-1} and 10^9 KW^{-1}. Apparently, these measured overall thermal resistances are generally 1-2 orders of magnitude larger than the estimated thermal contact resistances. As a consequence, the change of the thermal conductance of the suspended nanofiber determines the performance of the PE nanofiber thermal diodes.

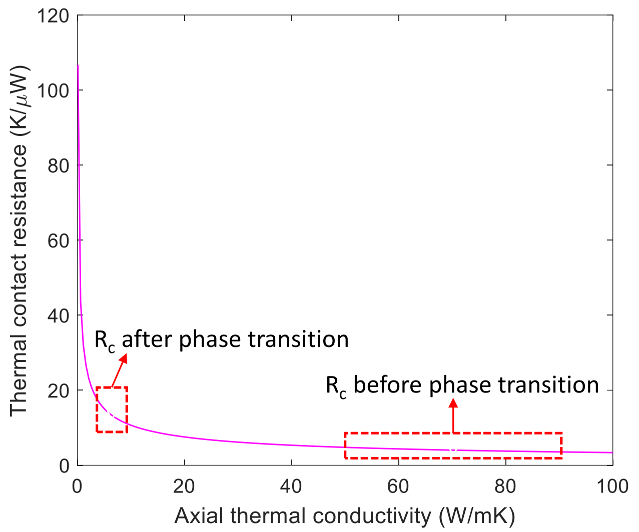

Supplementary Figure 8: Total thermal contact resistance between the PE nanofiber and two suspended islands as a function of the axial thermal conductivity of the PE nanofiber. The thermal contact resistance decreases with the increase in the axial thermal conductivity of the PE nanofiber.

Supplementary Note 8: Uncertainty Analysis

Here we employ the Wheatstone bridge technique developed in Ref. [12] to measure the performance of the nanoscale thermal diodes. In order to obtain a clear relation between heat flux and temperature bias, we measure the Q-ΔT relation through the Wheatstone bridge measurement. Provided that the input noise of the SR830 amplifier is 5 nV/$\sqrt{\text{Hz}}$ and the equivalent noise bandwidth (denoted as Δf) is around 0.3 Hz, the noise at the amplifier input can be

$$N_{amp} = \left(5\ nV/\sqrt{\text{Hz}}\right) \cdot \sqrt{\Delta f} = 2.74\ \text{nV} \quad \text{(S2)}$$

And the Johnson noise of each resistor is obtained by

$$N_{J,i} = \sqrt{4k_B T_i R_i \Delta f} \quad \text{(S3)}$$

where T_i is the temperature of the resistor R_i. And in our experiment $T_s = T_1 = 400K$, $T_3 =$

$T_4 = 300K$, $R_1 = 1.826\ \text{k}\Omega$, $R_s = 4.499\ \text{k}\Omega$, $R_3 = 3.05\ \text{k}\Omega$, $R_4 = 3.02\ \text{k}\Omega$. Therefore, the total voltage noise at lock-in input is the summation of all the above noise sources.

$$\Delta V_g = \sqrt{\sum_i N_i^2} \approx 16.5\ \text{nV} \tag{S4}$$

When the resistance value is much larger than the change in resistance, the change of R_s can be calculated as

$$\Delta R_s = \left(\frac{\Delta V_g}{V_s}\right)\frac{(R_s + R_2)^2}{R_2} \approx 15.5\ \text{m}\Omega \tag{S5}$$

Based on the measured temperature coefficient of resistance (TCR) of the platinum coil (0.002/K), the noise equivalent temperature (NET) can be obtained as

$$\text{NET} = \frac{\Delta R_s}{R_s}\frac{1}{\text{TCR}} \approx 1.92\ \text{mK} \tag{S6}$$

Then the noise equivalent conductance (NEG_s) can be calculated as follows.

$$\text{NEG}_s = G_b \frac{\text{NET}}{\Delta T_h - \Delta T_s} \approx 52.9\ \text{pW/K} \tag{S7}$$

Here, G_b is the conductance of the suspending beams (~135 nW/K), and $\Delta T_h - \Delta T_s$ is the temperature difference between the heating and sensing islands (~5 K).

The uncertainty of the temperature-dependent thermal conductance measurement has been elaborately discussed in our previous work Ref. [2]. In a similar approach, the uncertainty of heat flow measurements is considered. Heat flow is calculated by:

$$Q = G_s(\Delta T_h - \Delta T_s) = \frac{I^2 \gamma R_h \Delta T_s}{\Delta T_h + \Delta T_s} \tag{S8}$$

Where G_s is the thermal conductance of sample, I is the value of DC current, R_h is the resistance of heating island and γ is a coefficient for the aggregate resistance of heating island and supporting beam.

Apply the error propagation formula, the uncertainty of heat flow is calculated by:

$$\left(\frac{\delta Q}{Q}\right)^2 = \left(\frac{2\delta I}{I}\right)^2 + \left(\frac{\delta R_h}{R_h}\right)^2 + \left(\frac{\delta \Delta T_s}{\Delta T_s}\right)^2 + \left(\frac{\delta(\Delta T_h + \Delta T_s)}{\Delta T_h + \Delta T_s}\right)^2 \tag{S9}$$

The uncertainty of DC current is given by the parameters of current source, KEITHLEY model 6220 DC current source, which has a 0.01% uncertainty. Uncertainty of R_h and T_h is calculated based on methods in Refs. [2] and [10]. Uncertainty of R_s is calculated based on methods in Ref. [12]. As shown in Supplementary Fig. 9, the uncertainty of heat flow is very small in our measurements. Here, we also study the uncertainty of thermal rectification factor. Based on the definition of thermal rectification factor,

$$R = \frac{Q_{fwd} - Q_{rev}}{Q_{rev}} \tag{S10}$$

where Q_{fwd} is the heat flow of the forward bias and Q_{rev} is the heat flow in the reverse bias. Heat flow value is determined by the least-square linear fitting in a 1.2 K temperature bias range. For instance, the linear fitting is applied in the range (8.4 K, 9.6 K) to determine the heat flow value at 9K temperature bias.

The uncertainty in R can be calculated using error propagation as follows.

$$\frac{\delta R}{R} = \sqrt{\left(\frac{\sigma(Q_{fwd})}{Q_{fwd}}\right)^2 + \left(\frac{\sigma(Q_{rev})}{Q_{rev}}\right)^2} \tag{S11}$$

where $\sigma(Q_{fwd})$ and $\sigma(Q_{rev})$ are the fitting errors of Q_{fwd} and Q_{rev} from the linear fitting, respectively.

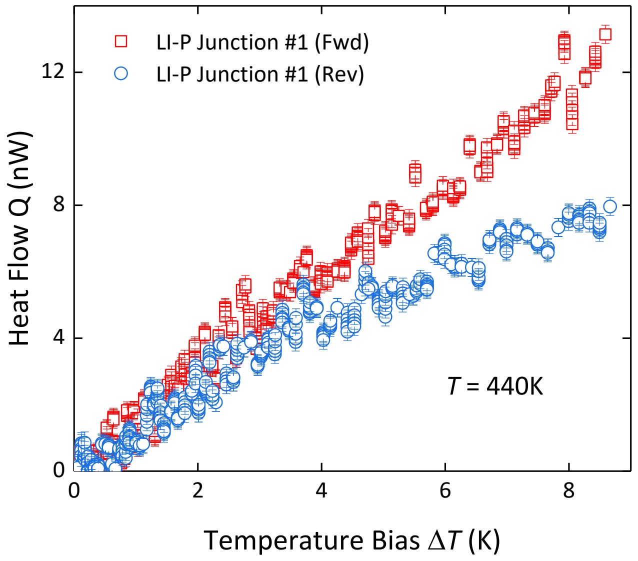

Supplementary Figure 9: Heat flow vs. temperature bias with error bars for LI-P nanofiber junction #1 at the working temperature of 440 K.

Supplementary Note 9: Thermal conductance of the heavily irradiated (HI) nanofiber sample

As shown in Supplementary Fig. 10, the structural phase transition disappears in a heavily irradiated (HI) PE nanofiber. The thermal conductance only slightly increases with temperature, which is similar to amorphous materials.

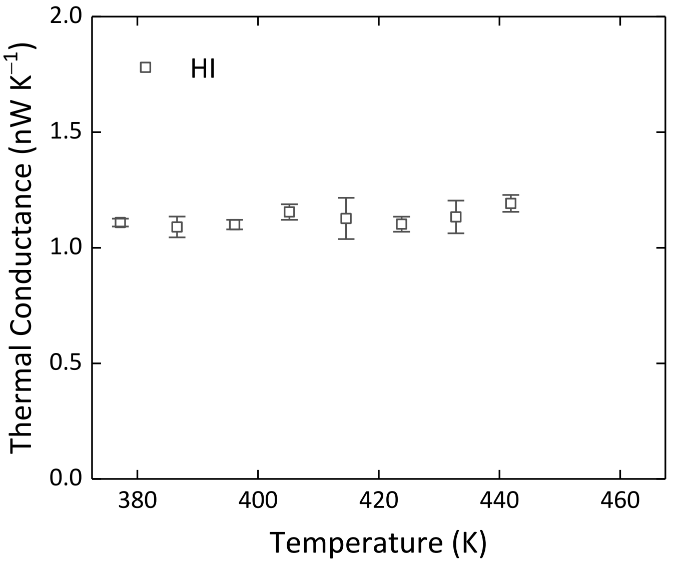

Supplementary Figure 10: Thermal conductance versus temperature of the HI PE nanofiber.

Supplementary Note 10: Background thermal conductance of a blank device due to thermal radiation

As shown in Supplementary Fig. 11, we measure the background conductance of a blank device due to thermal radiation at room temperature. The equivalent background thermal conductance is around 0.25 nW K^{-1}. As a comparison, the thermal conductances of all our samples are at least twice larger than 0.25 nW K^{-1} when rectification occurs, as shown in Supplementary Table 2. Background thermal conductance should be subtracted for the accurate measurement of thermal rectification ratio. In fact, the existence of background thermal conductance decreases the thermal rectification ratio. Thermal rectification ratio γ is defined as a ratio of heat flow at the same temperature bias, thus it can also be calculated by thermal conductance G:

$$\gamma = \frac{Q_{fwd} - Q_{rev}}{Q_{rev}} = \frac{G_{fwd}}{G_{rev}} - 1$$

The thermal conductance measured in our experiment is a combination of sample thermal conductance G_s and background thermal conductance G_{bg}:

$$G = G_s + G_{bg}$$

Take the HI-P junction as an example, as $G_{s\_fwd} > G_{s\_rev}$, mathematically

$$\frac{G_{s\_fwd} + G_{bg}}{G_{s\_rev} + G_{bg}} < \frac{G_{s\_fwd}}{G_{s\_rev}}$$

Thermal rectification ratio increases if background thermal conductance is subtracted.

Here, we assume a constant background thermal conductance 0.25 nW K^{-1} that was measured in Supplementary Fig. 11 and subtract the background heat flow $G_{bg}\Delta T$ to eliminate the influence of background thermal conductance. The rectification values without background thermal conductance are shown in Supplementary Table 3. Although such a subtraction could increase the thermal rectification ratio, background heat transfer cannot be eliminated in real-world applications of thermal diodes.

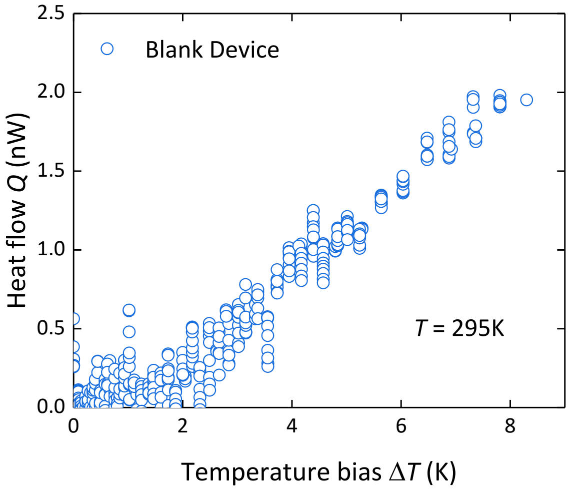

Supplementary Figure 11: Heat flow of a blank device at 295K.

Supplementary Table 2: Thermal conductances of all our samples

	Temperature (K)	Thermal conductance (nW K^{-1})
HI-P #1	435	~2.24
HI-P #2	448	~11.2
HI-P #3	435	~2.71
LI-P #1	390	~3.97
	440	~0.98
LI-P #2	395	~0.71
LI-P #3	405	~5.19

Supplementary Table 3: Rectification of all the samples with and without background thermal conductance

	Rectification w/ background	Rectification w/o background
HI-P #1	50.5 ± 3.6%	56.8 ± 4.0%
HI-P #2	48.2 ± 0.3%	53.6 ± 0.4%
HI-P #3	13.8 ± 0.6%	16.9 ± 1.0%
LI-P #1	−11.6 ± 1.3 %	−12.3 ±1.4%
	46.6 ± 6.1 %	62.6 ± 8.0%
LI-P #2	−11.5 ± 2.9%	−17.7 ± 4.5%
LI-P #3	−13.7 ± 3.5%	−14.4 ± 3.7%

Supplementary Note 11: Discussion about aging due to thermal cycles

Aging is a common problem of organic polymeric materials, and for our thermal diodes the rectification performance is expected to degrade when cycling between the highly-ordered phase and less-ordered phase. However, our cycling test shows the thermal diode based on the PE nanofiber can maintain an effective rectification within 20 cycles and > 24 h. Thus, we believe that the aging behavior of our current thermal diodes is acceptable to some extent.

References

1. Shen S., *et al*, Polyethylene nanofibres with very high thermal conductivities, *Nat. Nanotechnol.* **5**, 251–255 (2010).
2. Shrestha, R., *et al*, Crystalline polymer nanofibers with ultra-high strength and thermal conductivity. *Nat. Commun.* **9**, 1664 (2018).
3. Zhang, T. & Luo, T. High-contrast, reversible thermal conductivity regulation utilizing the phase transition of polyethylene nanofibers, *ACS Nano*. **7**, 7592-7600 (2013).
4. Zhang, T. & Luo, T. Giant thermal rectification from polyethylene nanofiber thermal diodes, *Small*, **11**, 4657-4665 (2015).
5. Bunn, B. C. W. The crystal structure of long-chain normal paraffin hydrocarbons. The "shape" of the <CH2 group, *Trans. Faraday Soc.* **35**, 482–491 (1939).
6. Tashiro, K., Sasaki, S. & Kobayashi, M. Structural investigation of orthorhombic-to-hexagonal phase transition in polyethylene crystal: the experimental confirmation of the

conformationally disordered structure by X-ray diffraction and infrared/Raman spectroscopic measurements, *Macromolecules*, **29**, 7460-7469 (1996).

7. Citra, M. J., *et al*, Molecular orientation of high-density polyethylene fibers characterized by polarized Raman spectroscopy, *Macromolecules*, **28**, 4007-4012 (1995).

8. Dames, C., Solid-state thermal rectification with existing bulk materials, *J. Heat Transfer*, **131**, 061301 (2009).

9. Shrestha, R., *et al*, High-contrast and reversible polymer thermal regulator by structural phase transition. *Sci. Adv.* **5**, eaax3777 (2019).

10. Shi, L., *et al*, Measuring thermal and thermoelectric properties of one-dimensional nanostructures using a microfabricated device, *J. Heat Transfer*, **125**, 881-888 (2003).

11. Yu, C., *et al*, Thermal contact resistance and thermal conductivity of a carbon nanofiber, *J. Heat Transfer*, **128**, 234-239 (2005).

12. Wingert, M. C., *et al*, Ultra-sensitive thermal conductance measurement of one-dimensional nanostructures enhanced by differential bridge, *Rev. Sci. Instrum.* **83**, 024901 (2012).
